# Prevalence and associated factors of herbal medicine use among breast cancer patients: a cross-sectional study in Morocco

**DOI:** 10.3332/ecancer.2024.1786

**Published:** 2024-10-09

**Authors:** Anass Baladi, Mohammed El Fadli, Hassan Abdelilah Tafenzi, Kawtar El Bouaouidi, Nada Benhima, Leila Afani, Ismail Essâdi, Rhizlane Belbaraka

**Affiliations:** 1 Department of Medical Oncology, Mohammed VI University Hospital of Marrakech, Marrakesh 40000, Morocco; 2 Laboratory of Biosciences and Health, Faculty of Medicine and Pharmacy of Marrakech, Marrakech 40000, Morocco; 3 Department of Medical Oncology, Avicenna Military Hospital of Marrakech, Marrakesh 40000, Morocco

**Keywords:** complementary and alternative medicine, integrative medicine, phytotherapy, breast cancer

## Abstract

**Background:**

Despite advances in modern medicine, an increasing number of breast cancer (BC) patients are turning to complementary and alternative medicine, such as phytotherapy. Instead of being prescribed by breast medical oncologists, patients are often seeking out phytotherapy themselves. They typically resort to herbal medicine as an alternative treatment to alleviate symptoms and side effects and enhance their quality of life during cancer treatment. This study, conducted in Morocco, aimed to investigate the prevalence and associated factors of herbal medicine use among BC patients.

**Methods:**

A cross-sectional study of 170 patients with BC was carried out from October 2021 to January 2022 at the Mohamed VI University Hospital in Marrakech. Participants were selected using convenience sampling based on specific criteria such as being over 18 years old, having a histological diagnosis of BC, and being in active treatment. Data were collected through a structured questionnaire administered by trained clinicians, and medical records were reviewed for clinical data. Statistical analysis was conducted using Microsoft Forms for data collection and SPSS version 26 for statistical analysis. Descriptive statistics summarised demographic and health-related characteristics. Associations between herbal medicine use and categorical variables were assessed using chi-square and Fisher exact tests. Logistic regression analyses were performed to identify predictors of herbal medicine use, with statistical significance set at *p* < 0.05.

**Results:**

Among the 170 BC patients included in the study, 37% reported using phytotherapeutics. One of the significant findings of this study was that nearly half of the BC patients surveyed believed herbal remedies to be harmless. None of the patients received information about herbal medicine use from their attending physicians. The use of herbal medication was significantly associated with marital status adjusted odds ratio (AOR: NR, *p* = 0.019), residence (AOR: 2.291, 95% confidence interval (CI): 1.214–4.324, *p* = 0.019), education levels (AOR: NR, *p* = 0.04) and receipt of radiotherapy (AOR: 0.128, 95% CI: 0.016–1.007, *p* = 0.023). Widowed women had a four times higher probability of using medicinal herbs than single or divorced women (AOR: 4.95, 95% CI: 1.16–20.90, *p* = 0.03). Illiterate women (AOR: 0.18, 95% CI: 0.052–0.65, *p* = 0.009) or those who attended Koranic school (AOR: 0.04, 95% CI: 0.004–0.47, *p* = 0.01) were less likely to use herbal medicine. Urban women were about twice as likely to use herbal remedies as women from rural areas (AOR: 2.02, 95% CI: 1.002–4.09, *p* = 0.049).

**Conclusion:**

This study highlights the need for healthcare professionals to be aware of their patients’ possible use of herbal medicine, be familiar with commonly used herbal treatments, and take proactive steps to explain any potential drug interaction and associated benefits. The findings of this study also provide insight into information on the sociodemographic and health-related factors associated with the use of herbal medicines among BC patients.

## Background

Breast cancer (BC) is one of the most substantial global health issues [1–4]. In women globally, it is the leading cancer by incidence with more cases diagnosed than lung cancer worldwide as of 2020 [1]. The disease makes up a huge proportion of cancer cases and deaths all over the world; almost 2.3 million new cases were reported last year along with 685,000 lives lost to it [2]. BC is still on the rise everywhere around the world which means that we are all affected differently based on where we live more advanced regions see higher survival rates while less developed ones struggle due to delays in diagnosis and ineffective treatment access [3]. The use of herbal medicine is considered very effective in combating the secondary effects that can result from conventional cancer treatment. According to research, herbal drugs such as licorice, astragalus, turmeric and others can prevent or weaken adverse reactions produced by chemotherapy and radiation therapy which helps improve the effectiveness of anti-cancer treatment as a whole [2, 5]. In a recent survey, 70% of cancer patients reported using complementary or alternative medicine (CAM) as part of their treatment plan [6]. In Spain, the use of complementary medicine (CM) among cancer patients is notably high, with studies indicating that approximately 81% of the general population has utilised some form of complementary therapy at least once [7]. Saudi studies found that the use of CAMs by cancer patients ranged between 25% and 80%, with natural products topping the list as the most commonly chosen type [8]. The prevalence of phytotherapy use among BC patients in Africa is notable, with traditional healers and practitioners incorporating medicinal plants in the treatment of BC due to factors like the high cost of conventional treatments and limited access to healthcare services [9–11]. The Moroccan population frequently practices phytotherapy. Studies have shown that, depending on the region, 55% to 90% of patients use plants to treat chronic diseases such as diabetes, hypertension and urinary infections [12].

Phytotherapy involves using plant-based medicines and supplements to treat illnesses and BC patients often use it to alleviate symptoms and the side effects of conventional cancer treatments. Studies have shown that phytotherapy, which involves the use of medicinal plants, is increasingly recognised for its potential in BC treatment, either alone or in combination with traditional therapies [9]. However, there is a substantial variation in prevalence, patterns, reasons for use and user characteristics across different geographic regions [13]. Many complementary therapies, particularly herbal remedies, lack controlled clinical trials to test their effects and some may interact with standard treatments and cause adverse effects. This poses a significant challenge for oncologists [14]. This study aimed to investigate the prevalence and associated factors of herbal medicine use among BC patients on standard treatment regimens in Morocco.

## Methods

### Study design and setting

We conducted a cross-sectional study between October 2021 and January 2022 in the medical oncology Department of Mohamed VI University Hospital in Marrakech, Morocco.

### Sample size and sampling technique

The sample size was not determined using a specific estimation formula, as we included all available and willing patients during the data collection period. Since we worked with all eligible patients who consented to participate in the study, this was not a random sample or sample size drawn, but rather a convenience sample based on the inclusion criteria and patient availability. There were two groups of patients: Phyto users and non-Phyto users.

### Inclusion and exclusion criteria

Participants had to be over 18 years old with a histological diagnosis of BC. Exclusion criteria included patients currently in the diagnosis process, those who refused the questionnaire or those in palliative care who were too weary to participate. Patients younger than 18 years were excluded from the study for several reasons. First, BC is sporadic among adolescents, meaning their inclusion could skew results and statistical analyses. Second, the ethical considerations and parental consent required to include minors in research studies can be complex and may limit the ability to collect reliable and consistent data.

### Data collection technique

We used a structured questionnaire to collect data on the use of herbal medicines among BC patients. However, we acknowledge that the questionnaire was not subjected to a formal local validation process specific to the Moroccan context. Instead, the questionnaire was adapted from previous studies with similar objectives and was reviewed by oncologists, to ensure cultural relevance and comprehensibility for the study population. Illiterate patients were assisted in completing the questionnaire to ensure accurate data collection. Information collected included sociodemographic variables, use of medicinal plants, reasons for their use, sources of information and patient perspectives on herbal versus synthetic medicines. Clinical parameters and follow-up data were obtained from medical records. In this study, patients were recruited from a diverse range of medical situations within the oncology department. These included patients undergoing curative treatment regimens as well as those with metastatic disease. The distinction between these groups was based on clinical assessments and staging according to the eighth edition of the American Joint Committee on Cancer TNM staging system.

### Operational definitions

Phyto users: Patients who reported using herbal medicines.

Non-Phyto users: Patients who did not report using herbal medicines.

Herbal medicines: Plant-based substances used for medicinal purposes.

### Data analysis procedure

Data collection was done using Microsoft Forms, and statistical analysis was performed using SPSS version 26. Descriptive statistics, including frequency counts, percentages and mean standard deviations for categorical data, were calculated. The relationship between herbal remedy usage and other demographic and health-related factors was assessed using chi-square and Fisher exact tests. We used univariate and multivariate logistic regression analyses to control for confounders in this study. These analyses allowed us to evaluate the impact of each independent variable on the use of herbal medicine while adjusting for other variables that could confound the results. Variables that showed a statistically significant correlation in the univariate analysis were included in the multivariate model to determine their independent effect on medicinal plant use. Additionally, patients’ sociodemographic and clinical data were carefully collected and considered to minimise potential bias. Statistically significant differences were defined as *p*-values less than 0.05.

## Results

### Sociodemographic and health-related characteristics

A total of 170 participants were selected in the medical oncology department, out of which 63 patients (37%) were identified as herbal medicine users compared to 107 patients (62.94%) who were not. Thirty patients (47.6%) had metastatic-stage cancer. All patients included in the study were female, with a mean age of 51.3 years (range: 25–75 years). The majority of patients fell between the ages of 40 and 70, with a standard deviation of 10.9 and a median of 52.5 (43.0; 59.0). All patients received treatments, including chemotherapy (63.5%, *n* = 108), targeted therapy (20.6%, *n* = 35), hormonal therapy (28.2%, *n* = 48) and radiotherapy (7.6%, *n* = 13), either alone or in combination.

Of the total respondents, 121 patients (71.17%) were married and the majority of respondents, 114 patients (67.1%), were illiterate. Ninety-two patients (54.1%) were from rural areas, and only six had medical insurance (Table 1).

### Association between herbal medicine use and sociodemographic and health-related factors

Table 2 displays the statistically significant associations between the usage of herbal medication and marital status adjusted odds ratio (AOR: NR, *p* = 0.019), residence (AOR: 2.291, 95% confidence interval (CI): 1.214–4.324, *p* = 0.019), education levels (AOR: NR, *p* = 0.04) and receipt of radiotherapy (AOR: 0.128, 95% CI: 0.016–1.007, *p* = 0.023).

### Predictors of herbal medicine use

Table 3 displays the results of both univariate and multivariate logistic regression analyses concerning predictors of herbal medicine usage among BC patients after treatment. The analysis revealed that widowed women (AOR 4.95, 95% CI 1.16–20.90, *p* = 0.03) had a four times higher probability of using herbal remedies compared to single or divorced women. In addition, illiterate women (AOR: 0.18, 95% CI: 0.052–0.65, *p* = 0.009) and those who attended Koranic school (AOR: 0.04, 95% CI: 0.004–0.47, *p* = 0.01) were less likely to use herbal medicine. Moreover, women from urban areas were twice as likely to use herbal remedies as women from rural areas (AOR: 2.02, 95% CI: 1.002–4.09, *p* = 0.049).

### Sources of information for herbal medicine

Table 4 shows the sources of information on the herbal medicines used among patients with BC. Almost half of the patients (63,49%,* n* = 40) obtained information from their direct entourage, while 38% (*n* = 29) obtained information through discussions with other patients. The use of medicinal plants was recommended by family or friends in 66.66% (*n* = 42) of the cases, while the patient’s own choice accounted for 36.50% (*n* = 23) of the cases. None of the patients in this study received information about herbal medicine use from their attending physicians.

### Types of plants

**Figure 1.**
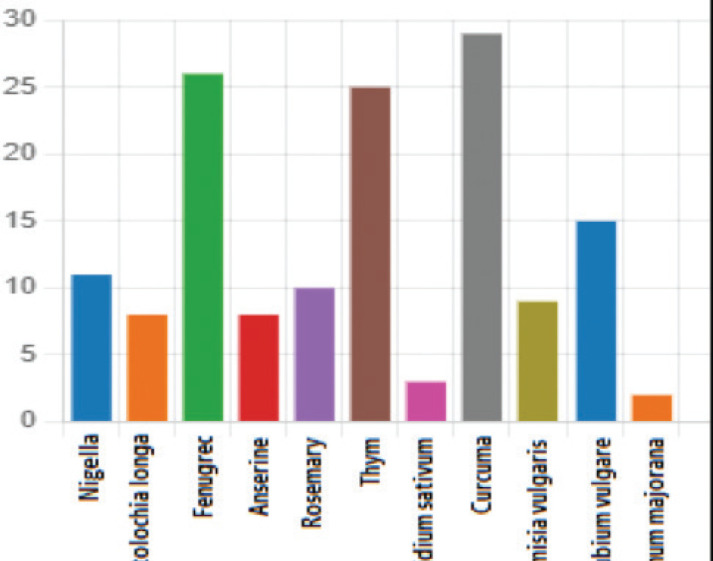
The different plant species used by BC patients at the Mohammed VI Oncological Center in Marrakech (October 2021–January 2022).

Thirteen different traditional substances were used by the patients. Twenty-nine patients (46.03%) used turmeric as an herbal medicine, while 26 patients (41.2%) used fenugreek. Additionally, 32 patients (50.7%) used a combination of plants. The specific plant species used by the patients are detailed in Figure 1.

### How patients take herbal medicine

Among patients, 33 (52.38%) began using phytotherapy before starting chemotherapy, 17 (26%) started during chemotherapy and 15 (23.8%) used it after completing chemotherapy, despite the potential risk of interaction with oncological treatment. Only six patients (10%) reported taking the herbal remedies daily (Table 4).

### Patients’ beliefs about herbal medicine

More than 50% of the patients believed that herbal remedies were harmless. Sixteen patients (25%) using phytotherapy believed that these plants have a synergistic effect with their treatment. Only 3% believed that herbal medicine is more effective than conventional treatment, while 31 patients (49%) believed it is safe. Twenty-four patients (38%) of patients believed that these plants are beneficial in alleviating the side effects of chemotherapy, with 27 patients (42%) reporting alleviation of pain and 7 patients (10%) reporting a decrease in nausea and vomiting. Five patients (7%) reported an improvement in diarrhea after using herbal medicine. Only one patient declared that she takes the plants to avoid the risk of neutropenia and to prevent postponing chemotherapy sessions. Twenty-two patients (35%) received sufficient information on the effectiveness and safety of herbal medicine before using it. 30 patients (43.47%) reported no benefit from using herbal medicine, while 8 patients (11.57%) reported a disappearance of pain and 5 patients (7.2%) reported feeling better and healthier after using it. Three patients reported a reduction in the size of their tumours (Table 4).

### Adverse effects

Six patients exhibited an impact on their liver function tests, and two of them required a dosage reduction according to the recommendations. Among the patients, 19 patients (31%) reported adverse effects, with weight loss being the most commonly reported at 11 patients (17%), followed by anorexia at 8 patients (11.5%) (Table 4).

## Discussion

Herbal remedies or phytotherapy, are commonly used by cancer patients worldwide as CAM. Patients often self-medicate with natural products made from whole or part of plants [15–17]. Although these remedies contain potentially active components, their interactions with conventional medications may increase side effects or decrease the effectiveness of BC treatments. This can lead to compromised treatment outcomes and increased health risks for patients [18].

Numerous studies have examined the prevalence and knowledge of phytotherapy among patients with BC. An evaluation report indicated patients from the United States, Europe, China and Australia. The prevalence rates ranged from 40.3% to 94.7%. Among the causes for this wide range noted by the authors are disparities in the definitions of CAM, different modalities of CAM, as well as variations in research methods (self-reporting or interviews) and geographic plus cultural factors together with attitude toward acceptance of CAM [19]. The rate of herbal medicine use among women with BC differs from one study to another. These studies show that a large number of women with BC use herbal products in addition to other therapies [9, 18, 20]. Brazilian cancer patients turn to herbal remedies up to 48.12%, attracted by the promise of relief from symptoms, among other benefits [21]. At the same time, a German study revealed that 30.1% of patients treated for breast or gynecological cancer by systemic therapy used herbal medicine in addition to conventional treatment [22]. A study conducted in Nigeria reports a prevalence of herbal medicine use of 34.5%, which is similar to the finding of 37%. Our study population had an average age of 51.3 years, consistent with previous studies in Fez [23] (49.1 years), France [21] (57.2 years), Nigeria [25] and others (51.9 years). These findings suggest that a significant number of BC patients use phytotherapy as a complementary therapy.

Phytotherapy may potentially improve or decrease the efficacy of conventional BC treatments. Among the plants used by the patients, curcumin was the most commonly used at 46.03% (*n* = 29). It is a natural compound found in turmeric, which is widely cultivated in Southeast Asia [26, 27]. Curcumin exhibits potential anti-cancer properties by influencing several biological mechanisms. A meta-analysis of randomised clinical trials indicates that curcumin effectively reduces TNF-α levels, a pro-inflammatory cytokine linked to chronic inflammation and various cancers. By lowering TNF-α, curcumin may help mitigate chronic inflammation, potentially slowing or preventing cancer progression driven by inflammation [28]. *In vitro* and animal studies reveal that curcumin can inhibit Stat3, a protein crucial for cancer cell growth and survival. It also suppresses matrix metalloproteinases, which degrade the extracellular matrix and facilitate cancer spread, and vascular endothelial growth factor, which supports tumour blood vessel formation [29, 30]. Curcumin promotes cancer cell apoptosis through caspase-dependent and mitochondrial pathways [31, 32]. Additionally, it negatively regulates cyclin-dependent kinases, which control cell cycle progression, thereby slowing cancer cell division [32]. In BC, curcumin might inhibit chemotherapy-induced apoptosis by blocking the c-Jun NH2-terminal kinase pathway and generating reactive oxygen species. This suggests curcumin could protect normal cells during chemotherapy, although more research is needed to fully understand its effects on cancer cells and its overall impact [33]. In an animal experimental study, the bioactive compounds in turmeric like curcumin C3 complex and bisdemethoxycurcumin have been shown to attenuate glial activation, oxidative stress and mitochondrial dysfunction, including mechanical hypersensitivity in neuropathic pain conditions [34]. Curcumin is known to inhibit cytochrome P450 enzymes, potentially affecting the metabolism of certain medications, though its clinical relevance remains unclear [35–38]. In a study of 60 BC patients, 2 g/day of curcumin caused only minor, clinically insignificant changes in paclitaxel pharmacokinetics [39, 40]. Additionally, curcumin inhibits doxorubicin-induced apoptosis in BC cell lines and impedes cyclophosphamide-induced tumour regression in animal studies, but the clinical significance of these effects is still unknown [33]. Turmeric has shown promise in relieving musculoskeletal pain. In a pilot, randomised clinical trial, Studies have highlighted the potential benefits of turmeric extracts for relieving knee joint pain [41, 42], with formulations containing water-soluble turmeric extracts demonstrating analgesic effects and potential inflammation reduction [42]. Additionally, A systematic review has explored the effects of turmeric on musculoskeletal pain, indicating a significant reduction in pain levels comparable to nonsteroidal anti-inflammatory drugs like ibuprofen [43].

The second most commonly used plant was fenugreek at 41.2% (*n* = 26). Fenugreek is an herbaceous plant of the Fabaceae family native to the Middle East and India. It is mainly used as a medicinal and condiment plant, and its active substance is isolated in seeds, which are still found in the form of seeds included in preparations. Fenugreek has been found to have anticancer effects, and its chemical components are known to cause apoptosis. A study by Khoja *et al* [44]. has demonstrated that MCF-7 breast cells can be effectively killed by chloroform seed extract, and according to Amin *et al* [45], this little plant has a protective effect on BC cells and prevents MDA 231 from inducing mammary hyperplasia. In our country, fenugreek is used as a tonic, for hair care, as a hypoglycemic and against heart palpitations. These findings suggest that phytotherapy may have synergistic effects with conventional BC treatments, potentially improving treatment outcomes. However, more clinical studies are needed to validate the effectiveness and safety of phytotherapy in combination with conventional treatments.

The univariate and multivariate logistic regression analyses of extracted variables related to phytotherapy usage among BC patients after treatment showed that widowed women (AOR 4.95, 95% CI 1.16–20.90, *p* = 0.03) had a four times higher probability of using herbal remedies than those who were single or divorced. In a Nigerian study, this percentage was close to our average (77.9%) [25]. Üstünda *et al* [46] investigated the relationship between civil status and the prevalence of the use of herbal medicines, but the results were statistically insignificant (*p* = 0.78). Research indicates that married/divorced women are more likely to use herbal medicine daily, with a 4.4-fold increase compared to unmarried individuals [47]. Widowed women tend to use herbal remedies more than those who are single or divorced due to various factors. Research shows widows are more independent and have a stronger work ethic. They also put in more hours and make all of the decisions regarding providing for their families [48]. Moreover, women are burdened by societal expectations, which makes them susceptible during widowhood especially when they do not have time for self-care [49]. Additionally, herbal medicines have traditionally been used to alleviate symptoms and improve the quality of life in different stages of women’s lives, including after menopause, which could contribute to the preference of widowed women for herbal remedies [50]. In general, the combination of cultural beliefs, perceived benefits and historical usage of herbal remedies likely influences widowed women to use these natural treatments more frequently.

In our study, it was found that illiterate women (AOR: 0.18, 95% CI: 0.052–0.65, *p* = 0.009) or those who attended Koranic school (AOR: 0.04, 95% CI: 0.004–0.47, *p* = 0.01) were less likely to use herbal medicine. A study in Istanbul found that highly educated individuals preferred herbal therapy more, highlighting a potential correlation between education level and herbal medicine use [51]. In Germany, where plant remedies are commonly used alongside conventional care, medical students showed a high knowledge and usage of phytotherapy, suggesting that education promotes herbal medicine use [52]. Illiterate women and those who attended Koranic schools may have less access to written information about herbal medicines, including their uses, benefits and potential risks. This limited access can reduce their likelihood of using phytotherapy [46]. Additionally, The use of herbal medicines is common among people seeking alternative treatments for BC, with significant belief in their effectiveness [52, 53]. Educated women can try herbal medicine because of its promising anti-cancer properties and the possibility of alleviating the side effects of conventional treatments [53]. According to other research reports, illiterate women who do not have access to or do not believe in current health systems generally receive herbal remedies based on recommendations from other locals or traditional herbalists, without scientific training [54]. The use of alternative medicine is generally more practiced by individuals with a low level of education, because they may have a negative view of conventional medicine (considering it contrary to alternative methods) or have the feeling that doctors do not support their preferred choice due to their noncompliance with alternative methods [51]. These discrepancies could be attributed to cultural differences, access to healthcare services and varying perceptions of herbal medicine across different regions.

The results of our study indicated that individuals residing in urban regions were more likely to utilise herbal medicine than those residing in rural areas (AOR: 2.02, 95% CI: 1.002–4.09, *p* = 0.049), which contrasts with the common assumption that individuals in rural areas would use them more frequently. These findings are consistent with those of previous studies [55,56]. Access to information is indeed higher in urban areas, potentially leading to increased awareness of herbal remedies among city dwellers [57]. Urban environments, characterised by cultural diversity, are more cosmopolitan and open to integrating herbal medicinal practices from various cultures [58]. Studies show that urban residents tend to have better access to health information through various channels such as social networks and interpersonal communication, which can influence their beliefs about herbal medicine [59]. However, there is a need for improved health education emphasising herbal products and socio-economic factors to promote herbal consumption among urban populations [60]. This highlights the importance of tailored strategies for urban areas to leverage their information access and cultural diversity for promoting herbal medicinal practices.

The primary source of information regarding phytotherapy was found to be the individual’s social circle, accounting for 74.3% of cases, as observed in a study conducted in Fez, as well as in subsequent studies in France, Nigeria and a European meta-analysis [24, 25, 61, 62]. Another significant source of information, as reported in an American study, was the Internet [63]. However, it is important to warn patients that not all information available in search engines can be relied upon. In Morocco, despite the increasing availability of internet access, our study found that it only accounted for 7.93% of the sources of information for patients.

BC patients often use phytotherapy to alleviate the side effects of conventional treatments, such as chemotherapy-induced nausea and vomiting (CINV), fatigue and pain. Based on the research provided, it is evident that ginger has been extensively studied for its effectiveness in reducing CINV in cancer patients [64–67]. Multiple randomised controlled trials have shown that ginger can significantly decrease the severity and frequency of CINV, without causing serious side effects, making it a safe option for managing this distressing side effect of chemotherapy [64–66]. Similarly, ginseng, another plant-based drug, has been found to improve fatigue in patients with BC who undergo chemotherapy [68]. Although some studies suggest that ginseng extracts may have estrogenic effects, particularly at low concentrations, which could potentially stimulate the growth of oestrogen receptor positive BC cells [69], other research indicates that ginseng can induce apoptosis in estrogen receptor-positive BC cells and enhance the effects of tamoxifen, a common treatment for this type of cancer [70]. Overall, the evidence is mixed, suggesting that some forms of ginseng may exhibit estrogen-like effects, while others might provide benefits in the treatment of hormone-sensitive BC.

In our study, 43.7% of patients reported no perceived benefit from using herbal medicine. These findings are consistent with a study conducted in Nigeria, which found that 58.3% of the patients reported a reduction in symptoms after using herbal medicine [71]. However, it is challenging to assess the effectiveness of phytotherapy since patients in our study used it alongside conventional treatment. Despite the potential benefits of phytotherapy in the treatment of BC, it is crucial to note that plant-based medicines and supplements can also have adverse effects and interact with conventional treatments. For example, St. John’s wort, a commonly used plant-based supplement, can interact with chemotherapy drugs, reducing their efficacy [72].

In our study, we observed the impact of herbal medicines on liver function tests in six patients and 31% (*n* = 19) reported experiencing adverse effects. The liver function of these patients improved after stopping herbal medicine. Among these, weight loss was the most frequently reported at 17% (*n* = 11), followed by anorexia at 11.5% (*n* = 8) [73]. These results are consistent with findings from previous studies. For instance, a study conducted in Japan found that 62.2% of patients did not experience adverse effects, while 5.3% reported side effects such as nausea, diarrhea, constipation, rash and liver dysfunction [74]. A European meta-analysis showed that only 4.4% of the patients reported side effects from complementary therapies, such as stomach upset and nausea, pruritus, headache and deterioration of kidney function [62].

This study highlights the significant prevalence of the use of herbal medicine by BC patients. Healthcare providers should be aware of this and discuss herbal medicines with their patients to ensure that they are taking care of them completely and that there are no other conventional treatments that interact with them. However, patients often do not inform their physician about their use of herbal medicine, as they may feel uncomfortable or uncertain about how their physician will react. This miscommunication between the oncologist and the patient often arises because the former is primarily concerned with the potential toxic effects and the risk that the patient may abandon conventional treatment in favour of CM, while the patient is more focused on the benefits of phytotherapy. Further research could focus on specific herbal remedies often used by women with BC, evaluating how they work, their risk profiles as well as possibilities for interaction with traditional therapies. Longitudinal surveys could also examine herb use treatment outcomes and quality of life in BC patients.

## Study limitations

In our cross-sectional study, the main objective was to assess the prevalence of herbal medicine use among BC patients, as well as the factors associated with this use. We collected information on the general use of herbal products, but due to the design of the study, it was difficult to obtain specific details on aspects such as dose, frequency and duration of use. The participants were chosen conveniently rather than through a random or representative process. The results of the study may not be generalisable to the entire population.

## Conclusion

The prevalence of phytotherapy in cancer patients is high, as shown by the results of our study and supported by data from the literature. The effectiveness and safety of phytotherapy in the context of BC treatment remain unclear. More research is needed to determine its potential benefits and risks. It is essential for BC patients using phytotherapy to seek advice from their healthcare providers and be aware of possible interactions with conventional treatments. In our study, more than half of patients believe that natural products are harmless, and there is an urgent need to educate patients about the potential risks associated with medicinal plants. As healthcare providers, it is crucial to discuss the use of CAM therapies with BC patients and provide evidence-based information to help them make informed decisions about their treatment options. Understanding these factors will help oncologists provide better care to BC patients and avoid potentially harmful interactions between herbal remedies and standard treatments.

## List of abbreviations

AOR, Adjusted odds ratios; BC, Breast cancer; CAM, Complementary and alternative medicine; CINV, chemotherapy-induced nausea and vomiting; COR, Crude odds ratios.

## Conflicts of interest

The authors declare that they have no competing interests.

## Funding

None.

## Ethics approval and consent to participate

The Ethics Review Committee of the Marrakech Faculty of Medicine and Pharmacy granted its approval for data access. It was not necessary to obtain the patient’s informed permission. Before analysis, patient records were anonymised to ensure confidentiality. All methods were performed by the relevant guidelines and regulations.

## Consent for publication

Not applicable.

## Availability of data and materials

The data sets used and/or analysed during the current study are available from the corresponding author on a reasonable request.

## Author contributions

Conception and design: Anass Baladi, Mohammed El Fadli, Hassan Abdelilah Tafenzi, Rhizlane Belbaraka, Ismail Essaadi

Statistical analysis: Anass Baladi, Hassan Abdelilah Tafenzi

Data interpretation: All Authors

Financial support: All Authors

Administrative support: Faculty of Medicine and Pharmacy, Cadi Ayyad University, Marrakech, Morocco & Medical Oncology Department, Mohammed VI University Hospital, Marrakech, Morocco, Bioscience and Health Laboratory.

Provision of study materials or patients: Anass Baladi, Hassan Abdelilah Tafenzi

Drafting: Anass Baladi

Review, revise and approve the manuscript: All authors.

## Figures and Tables

**Table 1.** Sociodemographic and health-related characteristics of BC patients at Mohammed VI Oncological Center, Marrakech (October 2021–January 2022).

Characteristics	Categories	*N* (Percentage)
**Use**	Nonuser	107 (63%)
	User	63 (37%)
**Age**	25–45 years	50 (29.4%)
	46–65 years	100 (58.8%)
	> 65 years	20 (11.8%)
**Marital status**	Married	121 (71.2%)
	Single or devoured	35 (20.6%)
	Widow	14 (8.2%)
**Residence**	Rural	92 (54.1%)
	Urban	78 (45.9%)
**Educational level**	Illiterate	114 (67.1%)
	Primary school	32 (18.8%)
	Secondary school	14 (8.2%)
	Koranic school	10 (5.9%)
	University	0 (0%)
**Employment status**	Unemployed	165 (97.1%)
	Employed	5 (2.9%)
**Medical cover**	Non	164 (96.5%)
	Yes	6 (3.5%)
**ECOG PS**	1	161 (94.7%)
	2	8 (4.7%)
	3	1 (0.6%)
**Disease stage**	Metastatic	78 (45.9%)
	localised + axillary lymph node	58 (34.1%)
	Localised	34 (20%)
**Therapeutic strategy**	Adjuvant	79 (46.5%)
	Palliative	69 (40.6%)
	Neoadjuvant	22 (12.9%)
**Chemotherapy**	Yes	108 (63.5%)
	Non	62 (36.5%)
**Hormonotherapy**	Non	122 (71.8%)
	Yes	48 (28.2%)
**Targeted therapy**	Non	135 (79.4%)
	Yes	35 (20.6%)
**Radiotherapy**	Non	157 (92.4%)
	Yes	13 (7.6%)

**Table 2.** Association between sociodemographic and health-related factors and herbal medicine use among BC patients at Mohammed VI Oncological Center, Marrakech (October 2021–January 2022).

Characteristics	Categories	Non-user (*n* = 107)	User (*n* = 63)	*p* value
**Age**	25–45 years	33 (30.8%)	17 (27%)	
	46–65 years	60 (56.1%)	40 (63.5%)	0.607
	> 65 years	14 (13.1%)	6 (9.5%)	
**Marital status**	Married	81 (75.7%)	40 (63.5%)	
	Single or devoured	22 (20.6%)	13 (20.6%)	0.019
	Widow	4 (3.7%)	10 (15.9%)	
**Residence**	Rural	66 (61.7%)	26 (41.3%)	0.01
	Urban	41 (38.3%)	37 (58.7%)	
**Educational level**	Illiterate	76 (71%)	38 (60.3%)	
	Primary school	18 (16.8%)	14 (22.2%)	
	Secondary school	4 (3.7%)	10 (15.9%)	0.04
	Koranic school	9 (8.4%)	1 (1.6%)	
	University	0 (0%)	0 (0%)	
**Employment status**	Unemployed	105 (98.1%)	60 (95.2%)	0.281
	Employed	2 (1.9%)	3 (4.8%)	
**Medical cover**	Non	105 (98.1%)	59 (93.6%)	0.126
	Yes	2 (1.9%)	4 (6.4%)	
**OMS**	1	104 (97.2%)	57 (90.5%)	
	2	3 (2.8%)	5 (8%)	0.128
	3	0 (0%)	1 (1.5%)	
**Disease stage**	Metastatic	48 (44.9%)	30 (47.6%)	
	localised+ axillary lymph node	40 (37.4%)	18 (28.6%)	0.429
	Localised	19 (17.8%)	15 (23.8%)	
**Therapeutic strategy**	Adjuvant	51 (47.7%)	28 (44.4%)	
	Palliative	39 (36.4%)	30 (47.6%)	0.197
	Neoadjuvant	17 (15.9%)	5 (7.9%)	
**Chemotherapy**	Yes	72 (67.3%)	36 (57.1%)	0.184
	Non	35 (32.7%)	27 (42.9%)	
**Hormonotherapy**	Non	74 (69.2%)	48 (76.2%)	0.325
	Yes	33 (30.8%)	15 (23.8%)	
**Targeted therapy**	Non	88 (82.2%)	47 (74.6%)	0.234
	Yes	19 (17.8%)	16 (25.4%)	
**Radiotherapy**	Non	95 (88.8%)	62 (98.4%)	0.023
	Yes	12 (11.2%)	1 (1.6%)	

**Table 3.** Predictors of herbal medicine use among BC patients at Mohammed VI Oncological Center, Marrakech (October 2021–January 2022).

Characteristic	Categories	COR (95% CL)	*p* value	AOR (95% CL)	*p* value
Age	25–45 years 46–65 years > 65 years	1 1.20 (0.39–3.68) 1.55 (0.55–4.38)	0.608 0.748 0.404	- - -	- - -
Marital status	Single or devoured Married Widow	1 0.83 (0.38–1.82) 4.23 (1.10–16.27)	0.033 0.653 0.036	1 0.92 (0.38–2.20) 4.94 (1.16–20.90)	0.034 0.862 0.030
Residence	Rural Urban	1 2.29 (1.21–4.32)	0.049	1 2.02 (1.002–4.09)	0.046
Educational level	Secondary Illiterate Koranic school Primary University	1 0.17 (0.053–0.59) 0.04 (0.04–0.42) 0.28 (0.074–1.08) -	0.012 0.005 0.008 0.65 -	1 0.18 (0.052–0.65) 0.04 (0.004–0.47) 0.36 (0.89–1.48) -	0.011 0.009 0.010 0.159 -
Employment status	Unemployed Employed	1 2.625 (0.42–16.15)	0.298	- -	- -
Médical cover	Non Yes	1 3.55 (0.63–20.01)	0.150	- -	- -
OMS	1 2 3	1 3.04 (0.70–13.19) -	0.332 0.137 1.000	- - -	- - -
Disease stage	Localised Localised + axillary lymph node Metastatic	1 0.57 (0.23–1.36) 0.792 (0.35–1.79)	0.431 0.209 0.575	- - -	- - -
Therapeutic strategy	Adjuvant Neoadjuvant Palliative	1 1.86 (0.62–5.60) 2.61 (0.86–7.89)	0.207 0.265 0.088	- - -	- - -
Chemotherapy	Non Yes	1 0.64 (0.34–1.23)	0.186	- -	- -
Hormonotherapy	Non Yes	1 0.70 (0.34–1.42)	0.326	- -	- -
Targeted therapy	Non Yes	1 1.57 (0.74–3.34)	0.236	- -	- -
Radiotherapy	Non Yes	1 0.12 (0.016–1007)	0.051	- -	- -

COR (95% CL): Crude odds ratio (95% Confidence Interval), *p* value: Probability value, AOR (95% CL): Adjusted odds ratio (95% Confidence Interval)

**Table 4.** Pattern and perception of herbal medicine use among BC patients at Mohammed VI Oncological Center, Marrakech (October 2021–January 2022).

Questions	Responses	*N* (%)
When did you use herbal medicine?	Before starting chemotherapy During chemotherapy After completing chemotherapy	33 (52.38%) 17 (26.98%) 15 (23.80%)
What is your source of information on herbal medicine?	Surroundings The other patients The press The attending physician The Internet	40 (63.49%) 29 (46.03%) 3 (4.76%) 0 5 (7.93%)
How did you decide to use this herbal medicine?	Recommended by family or friends It's your own will Recommended by a healthcare professional	42 (66.66%) 23 (36.50%) 1 (1.58%)
How do you use herbal medicine?	Auto medication Dietitian Charlatan	42 (66.66%) 5 (7.93%) 16 (25.93%)
Do you think these plants have a synergistic effect with the treatment?	Yes Non	16 (25.93%) 47 (74.60%)
Do you think herbal medicine is more effective than treatment?	Yes Non	2 (3.17%) 61 (96.82%)
Do you think herbal medicine is harmless?	Yes Non	31 (49.20%) 32 (50.72%)
Do you think these plants are beneficial in alleviating the side effects of chemotherapy?	Yes Non	24 (38.09%) 39 (61.90%)
If you answered ‘yes’. in which cases?	Pain relief Reduction of nausea and vomiting Avoidance of alopecia Stop diarrhea Weight gain Avoiding neutropenia	27 (42.85%) 7 (11.11%) 0 5 (7.93%) 0 1 (1.58%)
Did you get sufficient information on the effectiveness and safety of herbal medication before you started using it?	Yes Non	22 (34.92%) 41 (65.07%)
What are your findings after taking herbal medicine?	The patient feels better Disappearance of pain Feels healthy / heals Reduction of tumour mass No profits	16 (25.39%) 8 (12.69%) 6 (9.52%) 3 (4.76%) 30 (47.61%)
Have you noticed any side effects after using herbal medicine?	Yes Non	19 (30.15%) 43 (68.25%)
If yes. which ones?	To lose weight Anorexia Nausea and vomiting Weakness Faintness Diarrhea / mucoid stools Abdominal bloating	11 (17.46%) 8 (12.69%) 7 (11.11%) 4 (6.34%) 0 5 (7.93%) 1 (1.53%)
